# The rising influence of lipid metabolism in lung cancer: a global research perspective

**DOI:** 10.3389/fonc.2025.1562621

**Published:** 2025-03-31

**Authors:** Chaoqun Wang, Ming Lei, Wei Wang, Yuanyuan Jiang, Jiefeng Zhang, Yi Zhang, Bin Zhao, Wenyang Wang

**Affiliations:** ^1^ Department of Clinical Laboratory, The Affiliated Taian City Central Hospital of Qingdao University, Taian, China; ^2^ Department of Minimally Invasive Oncology, The Affiliated Taian City Central Hospital of Qingdao University, Taian, China; ^3^ Department of Intensive Care Medicine, The Affiliated Taian City Central Hospital of Qingdao University, Taian, China; ^4^ Department of Emergency Medicine, The Affiliated Taian City Central Hospital of Qingdao University, Taian, China; ^5^ Department of Gynecology, The Affiliated Taian City Central Hospital of Qingdao University, Taian, China; ^6^ Department of Pediatric Surgery, The Affiliated Taian City Central Hospital of Qingdao University, Taian, China

**Keywords:** lipid metabolism, lung cancer, bibliometric, visualized analysis, trend

## Abstract

**Background:**

Lung cancer is a prevalent malignant neoplasm globally and the leading cause of cancer-related mortality, posing a significant threat to human health and imposing a considerable societal burden. Researchers have recently focused more on lipid metabolism in lung cancer. However, to date, there has been no bibliometric analysis of lung cancer in relation to lipid metabolism. This study used bibliometric methods to analyze the link between lipid metabolism and lung cancer.

**Methods:**

Publications on lung cancer and lipid metabolism from 1995 to 2024 were sourced from the Web of Science Core Collection (WoSCC). The Microsoft Excel, R-bibliometrix, CiteSpace, and VOSviewer software were used to analyze and visualize the data.

**Results:**

In this study, a total of 535 publications were identified, with a marked increase in the number of publications observed post-2016. Both China and the United States exerted substantial influence in this domain. Notably, the Chinese Academy of Sciences and Huazhong University of Science and Technology have demonstrated leadership in various aspects of lipid metabolism research related to lung cancer. Professor Ana Ramirez de Molina and *Frontiers in Oncology* were the most productive authors and journals respectively. Besides, keywords like “lipid metabolism”, “lung cancer”, “expression”, “metabolism” and “growth” were central to current research and are expected to continue driving future trends in lung cancer and metabolism studies.

**Conclusions:**

Research on the relationship between lung cancer and lipid metabolism was still in its early stages. Targeting lipid metabolism in lung cancer represented a promising therapeutic strategy, as inhibiting key enzymes involved in lipid biosynthesis and uptake has the potential to impede cancer progression and mitigate drug resistance. This bibliometric study was the first to thoroughly summarize research trends and developments in this area over the past thirty years, providing scholars with updated insights and identifying future research directions.

## 1 Introduction

Lung cancer is one of the most common malignant tumors worldwide and the leading cause of cancer-related deaths, posing a serious threat to human health (1). The global burden of lung cancer was significant, with approximately 2.48 million new cases and 1.8 million deaths reported in 2022 alone (2, 3). Furthermore, lung cancer is the most diagnosed malignancy and the leading cause of cancer-related mortality worldwide. The incidence and mortality rates of lung cancer vary according to sex, geographic regions, and socioeconomic factors. Projections suggest that if current trends continue, the number of new cases could rise to 4.62 million, with 3.55 million deaths anticipated by 2050 (3).

Lipid metabolism has emerged as a significant research hotspot in the field of cancer biology, particularly in identifying new metabolic targets for lung cancer treatment (4). The alteration of lipid metabolism is a defining characteristic of lung cancer, significantly contributing to tumor development, progression, and therapeutic resistance (5). Recent research has underscored the significance of lipid metabolic pathways and their potential as targets for therapeutic intervention in lung cancer (6). By understanding the complex interactions between lipid metabolism and cancer biology, researchers can devise targeted therapies that leverage the metabolic susceptibilities of lung cancer cells. This strategy has the potential to enhance treatment outcomes and offer renewed hope for patients afflicted with lung cancer (7, 8).

Bibliometrics is an emerging method for analyzing literature, offering a systematic approach to understanding research trends and hotspots across various fields (9). Despite its growing application, there remains a gap in comprehensive bibliometric studies specifically focusing on the intersection of lung cancer and lipid metabolism. Consequently, conducting a bibliometric analysis focused on lung cancer and lipid metabolism provided valuable insights into the current research landscape, identified leading researchers and institutions, and highlighted emerging trends and future directions. Through the application of bibliometric methodologies, researchers could systematically evaluate the role of lipid metabolism in lung cancer and explore innovative therapeutic strategies designed to improve patient outcomes.

## 2 Data acquisition and methods

### 2.1 Data collection and processing

The data were collected from the Web of Science core collection Databases. The search terms were presented in the following manner: TS=((lipid metabolism or fatty acid metabolism) AND (Lung Cancer or Pulmonary Cancer)) and the time range from January 1, 1995 to December 31, 2024. As reported previously (10), these records were downloaded in text format, encompassing details such as the author, title, summary, and citation information. The above results were uploaded to CiteSpace v6.2.R2 (https://citespace.podia.com) and Microsoft Excel 2021 for further analysis (10).

### 2.2 Bibliometric analysis

VOSviewer(version 1.6.19), CiteSpace, and R (version 4.4.2) were used for the statistical computing and graphics. VOSviewer utilized data from the Web of Science Core Collection (WoSCC) to construct bibliometric maps, enabling a detailed and comprehensive analysis of these maps with consideration of collaborative data (11, 12). CiteSpace was designed to analyze the underlying knowledge structures within scientific literature and to visualize the collected data (12).

## 3 Results

### 3.1 Global trends of publication

**Figure 1:**
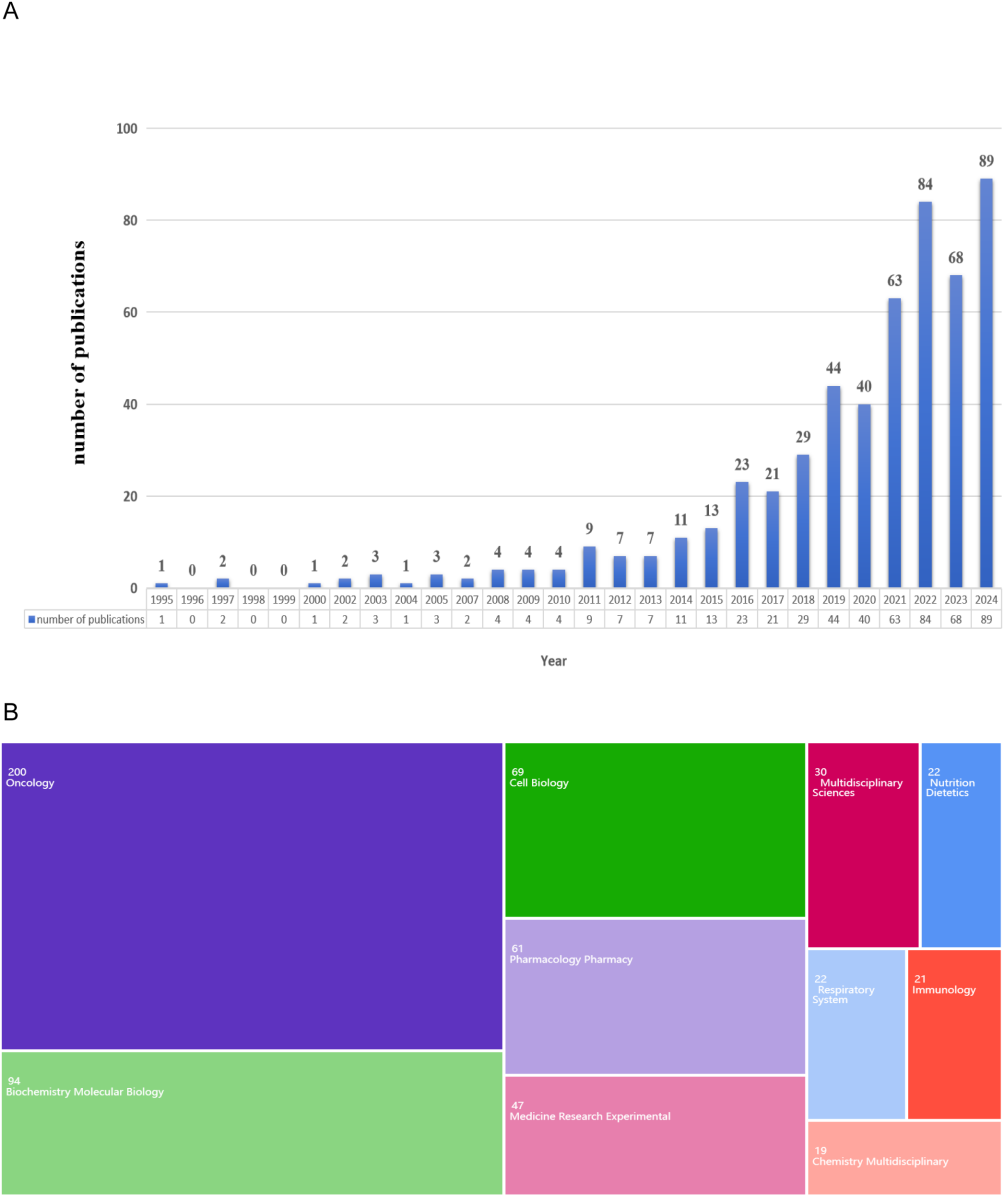
Global trends of publication. **(A)** Annual publications from 1995 to 2024. **(B)** Fields of the WoS categories associated with the lipid metabolism in lung cancer.

The annual publication count serves as a key indicator of the trend in the progression of scientific knowledge within a specific field. A total of 535 publications were rigorously selected based on predefined inclusion criteria (**Figure 1A**). Analysis of the temporal distribution of these publications revealed two distinct phases in the annual trend. From 1995 to 2015, the number of publications fluctuated within a narrow range of 0 to 20. Since 2016, there has been a significant upward trend in the number of publications. Notably, in 2024, the number of publications reached its highest point, totaling 89, which constitutes 16.64% of the total selected publications.

The focal point of this study can be elucidated through a comprehensive analysis of the research field. As illustrated in **Figure 1B**, the highest-ranked fields relevant to this investigation were highlighted. Specifically, the primary domains involved in this study were “Oncology” (200 publications), followed by “Biochemistry and Molecular Biology” (94 publications), and “Cell Biology” (69 publications).

### 3.2 Analysis of journals

**Table 1** listed the top 10 journals for co-citation in the field of lipid metabolism in lung cancer, with *Frontiers in Oncology* ranking first. This journal is renowned for its significant contributions to cancer research, focusing on publishing high-quality research articles and advancing the understanding of oncology. *Cancer Research*, which has a higher impact factor among oncology journals, highlights the critical importance and recognition of lipid metabolism in lung cancer as a key area of contemporary research.

**Table 1:** Top 10 journals for co-citation of lipid metabolism in lung cancer.

Rank	Journal	Number of publications	JCR	H-index	IF (2023)
1	FRONTIERS IN ONCOLOGY	20	Q2	136	3.5
2	CANCERS	16	Q1	133	4.5
3	INTERNATIONAL JOURNAL OF MOLECULAR SCIENCES	14	Q1	269	4.9
4	CANCER RESEARCH	12	Q1	498	12.5
5	SCIENTIFIC REPORTS	11	Q1	315	3.8
6	FRONTIERS IN PHARMACOLOGY	10	Q1	154	4.4
7	PLOS ONE	10	Q1	435	2.9
8	BMC CANCER	9	Q2	160	3.4
9	CANCER LETTERS	6	Q1	216	9.1
10	JOURNAL OF EXPERIMENTAL CLINICAL CANCER RESEARCH	6	Q1	120	11.4

### 3.3 Analysis of countries, institutions and authors

**Figure 2:**
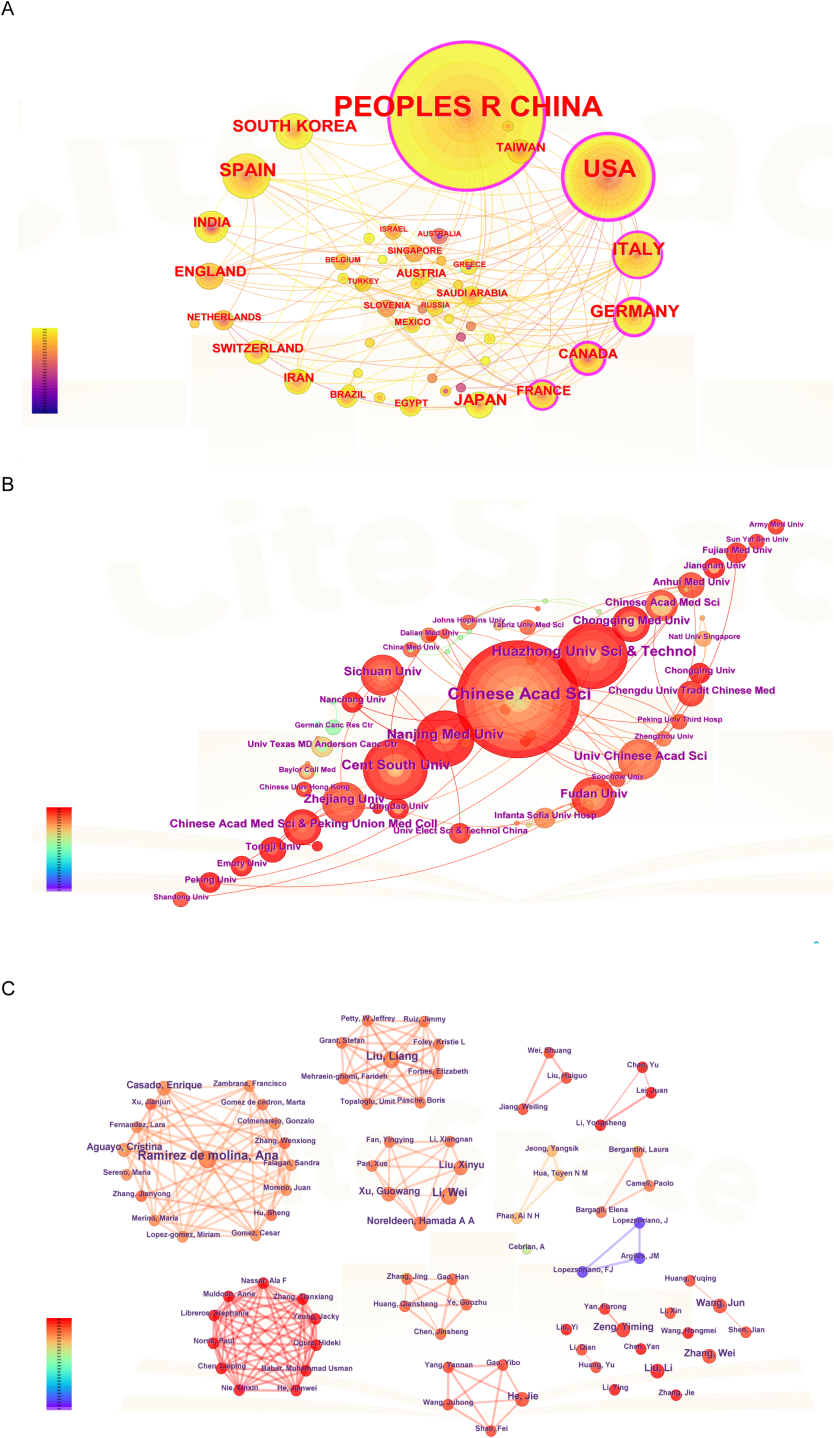
Analysis of countries, institutions and authors. **(A)** The visualization of countries. **(B)** The visualization of institutions. **(C)** The visualization of authors.

**Figure 2A** highlighted the collaborative efforts among a diverse range of countries and regions, particularly China, the United States, Italy, Germany, Canada, South Korea, Spain, Japan, and France. Furthermore, **Figure 2B** illustrated the institutional collaboration network, emphasizing the five most prolific Chinese institutions: the Chinese Academy of Sciences (CAS), Huazhong University of Science and Technology (HUST), Central South University (CSU), Nanjing Medical University (NJMU), and Sichuan University (SCU).

The authors’ collaboration network (**Figure 2C**) was mapped into six distinct clusters. In the network, each cluster represented a group of co-authors who predominantly originate from the same country or region. As illustrated in **Figure 2C**, most collaborative studies focused on clinical trials or cohort studies related to lipid metabolism in cancer. Notably, Professor Ana Ramirez de Molina, affiliated with the Madrid Institute for Advanced Studies on Food, is a distinguished expert in the field and has published five articles on lung cancer and lipid metabolism (13). Her research primarily centers on nutritional epigenetics in cancer. Similarly, Professor Wei Li from Chongqing University Cancer Hospital specializes in the role of energy metabolism in tumor cell death and has contributed four articles to this area (14, 15).

### 3.4 Analysis of cited articles

Utilizing VOSviewer, an analysis of the top ten most cited articles was presented in **Table 2**. Among these, three articles were published in the *Journal of Experimental Medicine*, while two appeared in *Signal Transduction and Targeted Therapy* and *Cancer Research*. The article with the highest citation count, authored by Xueli Bian in 2021 and titled “Lipid Metabolism and Cancer,” provides an extensive summary and discussion of the current understanding of lipid metabolism regulation in cancer cells. It also introduces various clinically employed strategies to disrupt lipid metabolism for cancer therapy (16). Meanwhile, the second most cited article, authored by Dingshan Li, presents an overview of the characteristics defining the interaction between ferroptosis and lipid metabolism, highlighting its significance in *Cancer Research* (17).

**Table 2:** Top 10 highly cited articles in the field of lipid metabolism in lung cancer.

Rank	Title	Author	Journal	Year	Total Citations	Average per Year
1	Lipid metabolism and cancer	Bian, Xueli	JOURNAL OF EXPERIMENTAL MEDICINE	2021	449	90.4
2	The interaction between ferroptosis and lipid metabolism in cancer	Li, Dingshan	SIGNAL TRANSDUCTION AND TARGETED THERAPY	2020	424	71.83
3	ATP Citrate Lyase: Activation and Therapeutic Implications in Non-Small Cell Lung Cancer	Migita, Toshiro	CANCER RESEARCH	2008	315	17.56
4	Lipid metabolism in cancer: New perspectives and emerging mechanisms	Broadfield, Lindsay A	DEVELOPMENTAL CELL	2021	303	61.2
5	Mechanisms of Metabolic Reprogramming in Cancer Cells Supporting Enhanced Growth and Proliferation	Schiliro, Chelsea	CELLS	2021	291	58.4
6	Lipids in the tumor microenvironment: From cancer progression to treatment	Corn, Kevin C	PROGRESS IN LIPID RESEARCH	2020	224	38
7	Fatty Acid Oxidation Mediated by Acyl-CoA Synthetase Long Chain 3 Is Required for Mutant KRAS Lung Tumorigenesis	Padanad, Mahesh S	CELL REPORTS	2016	215	21.6
8	Lipid metabolism and carcinogenesis, cancer development	Long, Jia	AMERICAN JOURNAL OF CANCER RESEARCH	2018	212	26.5
9	EGLN1/c-Myc Induced Lymphoid-Specific Helicase Inhibits Ferroptosis through Lipid Metabolic Gene Expression Changes	Jiang, Yiqun	THERANOSTICS	2017	211	23.44
10	Phospholipids and cholesterol: Inducers of cancer multidrug resistance and therapeutic targets	Kopecka, Joanna	DRUG RESISTANCE UPDATES	2020	183	30.67

### 3.5 Keywords and research trends

**Figure 3:**
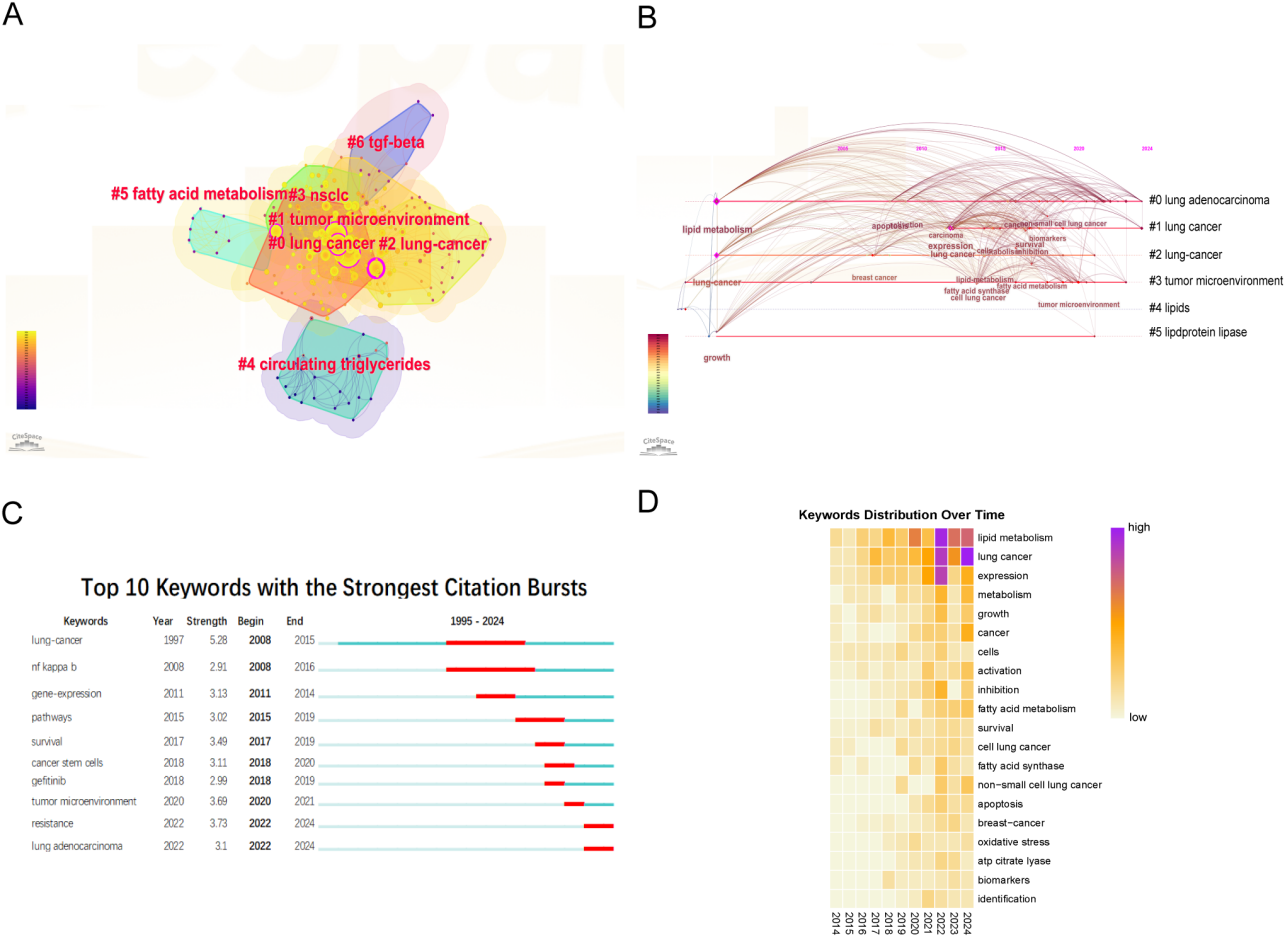
Analysis of keywords. **(A)** The clusters of keywords. **(B)** The timeline view map of keywords. **(C)** Burst detection of keywords. **(D)** Yearly occurrences of the top keywords.

Core keywords were selected using the traditional term frequency method (18). Subsequently, a total of 20 core keywords were classified into seven clusters, as illustrated in **Figure 3A**. These clusters encompass the following themes: “lung cancer,” “tumor microenvironment,” “lung-cancer,” “NSCLC,” “circulating triglycerides,” “transforming growth factor-beta (TGF-β),” and “drug resistance.” To elucidate the chronological progression of keywords, a timeline graph was utilized, resulting in the identification of six distinct natural clusters of keywords (**Figure 3B**). Notably, the clusters centered around ‘tumor microenvironment,’ ‘lipids,’ and ‘lipoprotein lipase’ emerged as focal points of research interest.

Additionally, a citation burst analysis of the top 10 keywords in the field were visualized (**Figure 3C**). Citation burst analysis is a methodological tool that identifies periods of significant increases in the frequency of specific terms or phrases within a research area, thereby assisting researchers in pinpointing emerging trends and hotspots (19). The keyword “lung cancer” (2008–2015) received the most sustained attention. However, recent years have seen an increasing focus on keywords such as “tumor microenvironment” (2020–2021), “drug resistance” (2022–2024), and “lung adenocarcinoma” (2020–2024). This trend suggesting that future research is likely to concentrate on these emerging areas. In addition, the analysis of keywords illustrated the focus on lipid metabolism in lung cancer research. In **Figure 3D**, the color gradient denotes the frequency of keyword occurrence, with more intense red hues signifying higher frequencies. Consequently, the most prevalent keywords in recent years comprised of “lipid metabolism,” “lung cancer,” “expression,” “metabolism,” and “growth”. These findings suggested that these terms constitute current focal points of research and were anticipated to persist as principal areas of interest and activity within the domains of lung cancer and metabolism. Besides, we conducted a search in the original database using the terms “omics” and “machine learning” and created a keyword map and a timeline graph. The timeline graph showed that studies related to “omics” and “machine learning” have concentrated since 2020, with a primary focus on lipid metabolism (including fatty acid metabolism) and growth. Notably, “omics” appeared in the abstract but was not listed as a direct keyword (**Supplementary Figure 1**).

## 4 Discussion

This study represents the pioneering application of bibliometric analysis to investigate the relationship between lung cancer and lipid metabolism. We systematically collected and examined various bibliometric indicators, including the number of publications, contributing institutions and regions, leading authors, and frequently occurring keywords. This research offers a comprehensive overview of current research trends and advancements in the domain of lung cancer and lipid metabolism.

In accordance with global publication trends, research into the relationship between lung cancer and lipid metabolism has deepened progressively over the past decade. Lipid reprogramming is closely linked to the epidermal growth factor receptor (EGFR) signaling pathway, which plays a crucial role in cancer development and progression (20–22). Understanding these mechanisms has unveiled potential therapeutic strategies targeting lipid metabolism for lung cancer treatment (22). In 2019, advancements in lipidomics and metabolomics technologies led to a significant surge in scholarly articles on lipid metabolism (23, 24). This demonstrates how technological innovation can drive scientific progress. Moreover, global research trends in nano-drug delivery systems for lung cancer have highlighted the importance of lipid nanoparticles as emerging hotspots (25). This may be another major reason for the increase in publications after 2019. These lipid nanoparticle systems offer promising strategies for optimizing drug delivery and overcoming drug resistance, further underscoring the critical role of lipid metabolism in lung cancer research and treatment (26).

The analysis of national and regional publication trends highlighted that China and the United States function as leading research centers across various scientific disciplines. Research institutions such as the CAS and HUST have emerged as significant contributors to lung cancer research, particularly in the context of advancements in metabolism regulation and extracellular vesicles. CAS is recognized for its prolific output and collaboration with other leading research centers globally. In the field of metabolomics, CAS has been instrumental in identifying novel recurrence-associated lipid metabolism signatures in early-stage lung adenocarcinoma (27). Specifically, the development of phospholipid phosphatase 2 (PLPP2)-mediated lipid raft synthesis represents an important biological event in the early progression of lung adenocarcinoma, providing potential targets for more precise diagnosis and treatment in clinical settings (28). Moreover, HUST has made substantial contributions to the study of extracellular vesicles in lung cancer. They conducted a metabolomic investigation of urinary extracellular vesicles for early detection and screening of lung cancer. The marker panel demonstrated effective prediction with an area under the curve (AUC) value of 84% (29).

Professor Ana Ramirez de Molina’s research focuses on the nutritional epigenetics of tumors while targeting cancer metabolism through lipid metabolism in cancer treatment and prognosis (30). This approach is supported by studies that underscore the critical role of lipid metabolism in cancer stem cells, which are well-known for their resistance to conventional therapies and their involvement in tumor recurrence (31). By integrating insights from nutritional epigenetics and cancer metabolism, her research enhances our understanding of how metabolic pathways can be modulated to improve cancer treatment outcomes.

Ferroptosis has been identified as a pivotal pathway in lung cancer, closely associated with lipid metabolism. This process is regulated by lipid metabolism, redox homeostasis, and epigenetic modifications, providing new avenues for precision therapy and overcoming drug resistance. For instance, hydrogen sulfide-induced persulfidation modulated homocysteine metabolism and potentiated ferroptosis in non-small-cell lung cancer (32). Furthermore, the combination of ferroptosis inducers with immune checkpoint inhibitors had the potential to enhance therapeutic outcomes by modulating the tumor immune microenvironment (33).

Keyword analysis revealed that lipid metabolism played a crucial role in the development of drug resistance in lung cancer (34). This association was based on the complex interaction between lipid metabolic pathways and cancer cell survival mechanisms. Alterations in lipid metabolism within cancer cells could lead to increased lipid synthesis or uptake, thereby facilitating rapid cell proliferation and contributing to drug resistance. For instance, the reprogramming of lipid metabolism has been implicated in resistance to therapies targeting the epidermal growth factor receptor (EGFR) in non-small cell lung cancer (NSCLC) (35). Additionally, modulation of lipid metabolism by drugs such as Anlotinib has been demonstrated to influence cancer cell sensitivity, suggesting that targeting lipid metabolic pathways could enhance the efficacy of existing treatments (36). Besides, lipid metabolism could also serve as a potential biomarker for diagnosing lung cancer. Tang et al. conducted the largest prospective untargeted metabolomics analysis in the Cancer Prevention Studies cohorts, identifying sphingomyelin (d18:0/22:0) and taurodeoxycholic acid 3-sulfate as metabolites positively associated with lung cancer risk, particularly stronger within three years of diagnosis. These metabolites may serve as potential screening biomarkers for lung cancer (37).

The tumor microenvironment was also highlighted in the keywords, with the interaction between lipid metabolism and immune responses playing a critical role in influencing the effectiveness of immunotherapies. Lipid metabolic reprogramming can modulate immune cell infiltration and function, thereby impacting the response to immune checkpoint inhibitors in lung cancer (38). This underscores the potential of targeting lipid metabolism not only to overcome drug resistance but also to improve the outcomes of immunotherapy (38, 39). Clinically, many anti-tumor drugs targeting lipid metabolism have emerged, and some have shown remarkable anti-tumor effects. The main issue now is enhancing the specificity of these inhibitors while maintaining normal cellular metabolism (40).

Nevertheless, this study is subject to several limitations. Primarily, the literature review was limited to sources available in the Web of Science Core Collection (WoSCC) database, which may have resulted in the omission of pertinent articles indexed in other databases. Additionally, the inclusion criteria were not exhaustive, potentially excluding relevant studies that could have enriched the analysis and provided a more comprehensive understanding of the subject matter. The reliance on a single database, such as WoSCC, inherently limits the breadth of the literature review. a more rigorous methodology would involve querying additional databases, including PubMed, Scopus, and Embase, to ensure a more comprehensive collection of relevant studies. This approach would mitigate the risk of overlooking critical studies and offer a more balanced and accurate representation of the existing body of research.

## 5 Conclusion

This study utilized bibliometric analysis to visualize research articles on lung cancer and lipid metabolism published between 1995 and 2024, identifying key trends, focal areas, and emerging topics. The findings indicate a notable increase in publications, with a significant contribution from China and the United States. Lipid metabolism is essential for meeting the energy and structural requirements of cancer cells, influencing signaling pathways that govern cell survival and proliferation. Additionally, it plays a pivotal role in the development of drug resistance in lung cancer.

## Data Availability

The original contributions presented in the study are included in the article/**Supplementary Material**. Further inquiries can be directed to the corresponding authors.
