# Supplementary material for: The rising influence of lipid metabolism in lung cancer: a global research perspective
Source: Front Oncol. 2025 Mar 31;15:1562621. doi: 10.3389/fonc.2025.1562621 (PMC11995272; doi:10.3389/fonc.2025.1562621)
Supplement: Supplementary file 1 [file DataSheet1.docx]

Supplementary Material

# Supplementary Figures and Tables

##
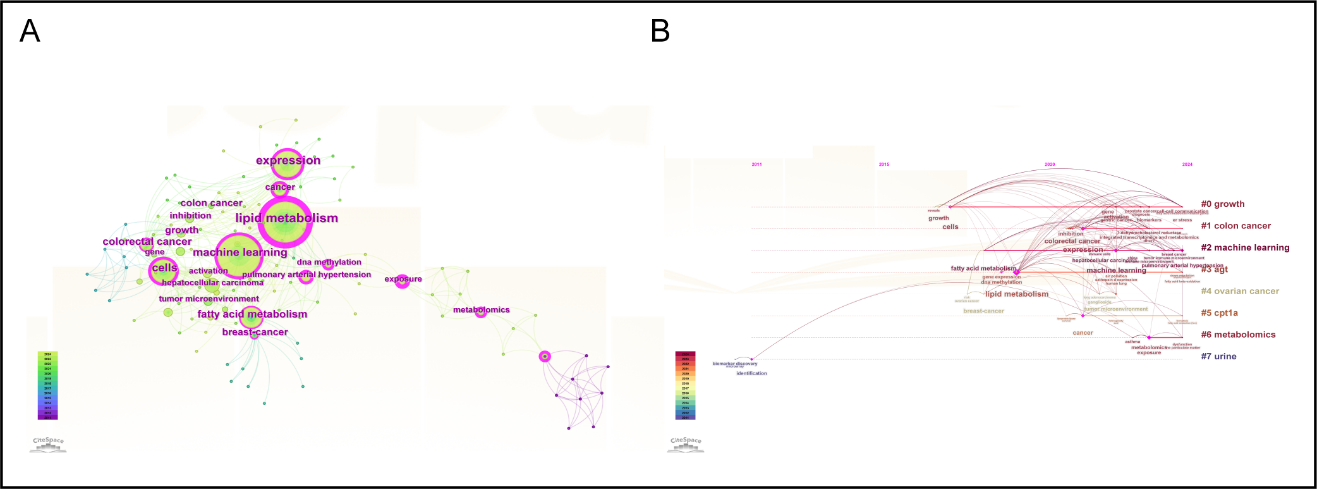
Supplementary Figures

**Supplementary Figure1.** Analysis of Subsets of Keywords (A) Cluster diagram of Keywords subsets, (B) Timeline view of Keywords subsets**.**
